# Implications of Lead (Pb)-Induced Transcriptomic and Phenotypic Alterations in the Aged Zebrafish (*Danio rerio*)

**DOI:** 10.3390/toxics12100745

**Published:** 2024-10-14

**Authors:** Chia-Chen Wu, Danielle N. Meyer, Alex Haimbaugh, Tracie R. Baker

**Affiliations:** 1 Institute of Environmental Engineering, National Yang Ming Chiao Tung University, 1001, Daxue Rd, East District, Hsinchu City 300093, Taiwan; cchenwu@nycu.edu.tw; 2 Department of Environmental and Global Health, University of Florida, 1225 Center Drive, Gainesville, FL 32610, USA; danielle.meyer@ufl.edu (D.N.M.);; 3 Department of Pharmacology, School of Medicine, Wayne State University, 540 E. Canfield, Detroit, MI 48201, USA; 4 UF Genetics Institute, University of Florida, 2033 Mowry Road, Gainesville, FL 32610, USA

**Keywords:** aging, movement disorders, nonmonotonic response, heavy metal

## Abstract

Lead (Pb) is a well-known neurotoxin with established adverse effects on the neurological functions of children and younger adults, including motor, learning, and memory abilities. However, its potential impact on older adults has received less attention. Using the zebrafish model, our study aims to characterize the dose–response relationship between environmentally relevant Pb exposure levels and their effects on changes in behavior and transcriptomics during the geriatric periods. We exposed two-year-old zebrafish to waterborne lead acetate (1, 10, 100, 1000, or 10,000 µg/L) or a vehicle (DMSO) for 5 days. While lower concentrations (1–100 µg/L) reflect environmentally relevant Pb levels, higher concentrations (1000–10,000 µg/L) were included to assess acute toxicity under extreme exposure scenarios. We conducted adult behavior assessment to evaluate the locomotor activity following exposure. The same individual fish were subsequently sacrificed for brain dissection after a day of recovery in the aquatic system. RNA extraction and sequencing were then performed to evaluate the Pb-induced transcriptomic changes. Higher (1000–10,000 ug/L) Pb levels induced hyperactive locomotor patterns in aged zebrafish, while lower (10–100 ug/L) Pb levels resulted in the lowest locomotor activity compared to the control group. Exposure to 100 µg/L led to the highest number of differentially expressed genes (DEGs), while 10,000 µg/L induced larger fold changes in both directions. The neurological pathways impacted by Pb exposure include functions related to neurotransmission, such as cytoskeletal regulation and synaptogenesis, and oxidative stress response, such as mitochondrial dysfunction and downregulation of heat shock protein genes. These findings emphasize a U-shape dose–response relationship with Pb concentrations in locomotor activity and transcriptomic changes in the aging brain.

## 1. Introduction

Understanding the impact of environmental exposures on the health of aged populations has become a pressing concern as our society undergoes a demographic shift towards older age groups. Currently, the number of people over the age of 60 surpasses that of children younger than 5 years old [1]. Aged populations are particularly vulnerable to the effects of environmental contaminants due to age-related changes in their physiologic, biochemical, and immunological systems [2]. Exposure to environmental contaminants can lead to earlier disease onset in older adults, with a particular susceptibility to neurological disorders [3].

Among the environmental toxicants of concern, lead (Pb) is a well-documented public health risk, known to impair motor, learning, and memory functions [4,5,6,7]. The rapidly expanding aged population has been historically exposed to high environmental lead levels due to early-life exposure from sources like gas, paints, solids, and dust in the 1960s–1980s. During this period, approximately 2% of NHANES-surveyed individuals reported blood Pb levels exceeding 30 ug/dL, which is about eight times the current blood lead reference value [8,9]. While early exposure can result in the accumulation of Pb in bones, multiple release events can occur during bone turnover and degredation throughout the aging process. As individuals age, the encephalic barrier weakens, allowing neurotoxicants such as Pb to readily cross the blood–brain barrier, which raises significant concerns about neurotoxic effects during aging.

The epidemiological evidence regarding the effects of low level Pb exposure on the elderly remains inconclusive. Some studies have indicated that bone Pb level, which reflects cumulative exposure, is associated with accelerated cognitive decline at older age [10,11]. However, concurrent blood Pb levels, which reflect more recent exposure, have an inconsistent relationship with neurological functions in adults [10,12,13,14]. It is worth noting that factors such as unknown exposure durations, variation in neurological indexes and endpoints, and survival bias among subjects can limit the conclusiveness of epidemiological studies regarding the impacts of low dose Pb exposure in older adults [11]. While animal models have explored Pb effects at various developmental stages [15,16,17] and in young individuals [18], there is a need for more research to understand the health effects of low-dose Pb exposure in aged populations.

To investigate the effects of Pb on aged populations, we used zebrafish as a model to study how environmental exposure influences the aging process. Zebrafish and humans share common functional characteristics associated with senescence, such as cognitive decline [19], osteoarthritis [20], and genome instability [21]. Starting at one year of age, zebrafish display signs of genomic instability, DNA fragmentation, and epigenetic alterations in somatic tissues [22]. Global genome methylation decreases in zebrafish aged 1, 1.5, and 2.5 years compared to younger individuals [21], and telomere length begins to shorten from 1.5 years onwards [23].

In our study, we conducted acute exposure experiments on zebrafish at 2 years of age to model older human adults. We assessed behavioral and transcriptomic responses to varying levels of Pb, including environmentally relevant concentrations (1–100 µg/L) [24,25] as well as higher levels (up to 10,000 µg/L) to evaluate potential acute toxicity scenarios. Our findings highilight both phenotypic effects and underlying dysregulation attributed to transcriptomic changes. These insights contribute to understanding the impact of neurotoxicants on aging trajectories and may inform the development of prevention strategies for age-related conditions.

## 2. Materials and Methods

### 2.1. Animals

Zebrafish (AB wildtype fish line) were maintained in a recirculating aquatic housing system in the vivarium space. Lab personnel provided routine animal care, and all procedures involving animals were approved by the Animal Care and Use Committee at Wayne State University and adhered to the NIH’s “Guide for the Care and Use of Laboratory Animals”. Zebrafish were raised according to our lab husbandry protocol (protocol number 19-02-0938) and procedures described by Westerfield [26].

### 2.2. Five-Day Acute Pb Exposure

Two-year-old zebrafish were exposed to waterborne lead acetate (Sigma-Aldrich, Saint Louis, MO, USA) at 0, 1, 10, 100, 1000, and 10,000 μg/L concentrations, or a vehicle (0.1% DMSO, *v*/*v*) for 5 days. For each concentration, four aged zebrafish were exposed. The exposure was conducted in a 1.5 L beaker with 800 mL exposure water. During exposure, 80% water changes with new exposure solution were performed every day. Since multiple studies have shown that Pb-induced behavioral impairment is sex-dependent, occurring mostly in males [27,28,29], our study focused on aged male zebrafish.

### 2.3. Adult Behavior Assessment

The behavior assessment was conducted to evaluate the neurological outcomes after 5-day acute Pb exposure between 13:00 and 17:00. An individual fish was placed into a novel tank filled with fish system water for 1 min to acclimate to the tank environment. Three-dimensional swimming behavior was recorded by two cameras positioned at the top and side of the tank. The distance travelled was tracked every 1/60 s by the video-tracking system (DanioVision, Noldus Information Technology, Wageningen, The Netherlands) over a 5 min period. We evaluated anxiety-like behaviors, including the number of times the zebrafish entered the top of the tank, duration in the top/bottom, distance traveled in top/bottom, erratic movements, and freezing bouts. The erratic movments were defined as instances where the fish exhibited velocities exceeding the 99th percentile of the control velocity distribution. Freezing bouts were defined as periods of total absence of movement lasting for 2 s. We also evaluated the velocity distribution by aggregating data points into 3 s intervals, resulting in 100 data points per fish replicate. The behavioral data statistics were analyzed by Kruskal–Wallis one-way analysis of variance and the Dunn–Bonferroni post–hoc test using R software (version 4. 2. 3, https://cran.r-project.org/bin/windows/base/old/, accessed on 16 February 2024). After the behavioral assays were finished, the fish was returned to the main aquatic housing system for a day until euthanization for RNA extraction.

### 2.4. RNA Extraction

To minimize noise and variability in the RNA-seq data, we collected three male adult fish replicates from each of the five exposure groups for transcriptomic analyses. The adult fish were euthanized in 16.7 mg/mL tricaine methanesulfonate solution. The brain was dissected and was saved in RNALater^TM^ (Thermo Fisher, Waltham, MA, USA) at −80 °C until RNA isolation. Before RNA isolation, RNALater^TM^ was removed from the tissue. Total RNA extraction was performed using the Quick-DNA/RNA^TM^ Miniprep plus Kit (Zymo, Irvine, CA, USA) following the manufacturer’s specifications. Briefly, each individual brain sample was pretreated with 95 μL DNase/RNase-Free Water, 95 μL PK Digestion Buffer, and 10 μL Proteinase K provided in the kit. The solutions were then incubated at 55 °C for 30 min, followed by a subsequent incubation at 94 °C for 20 min. After the incubation period, the solutions were centrifuged at 10,000× *g* for 30 s. The supernatant was transferred and mixed with DNA/RNA Lysis Buffer in a 1:1 ratio, after which they were transferred to the spin-columns for RNA and DNA purification following the manufacturer’s instruction.

### 2.5. Transcriptomic Sequencing and Analysis

A total of 15 brain RNA samples, comprising triplicates of five different exposure concentrations, were submitted for transcriptomic sequencing and analysis. Including five concentration groups in the experimental design has been shown to reduce the false discovery rate in differential gene expression analyses [30]. Before sequencing, the amount of isolated RNA was quantified using the Qubit 3.0 Fluorometer (Life Technologies, Darmstadt, Germany). RNA libraries were generated using the QuantSeq 3′ mRNA-Seq Library Prep Kit FWD for Illumina (Lexogen, Vienna, Austria) and subsequently subjected to sequencing at the Genome Sciences Core of Wayne State University. The libraries were processed on the Agilent TapeStation 2200 (Agilent Technologies, Santa Clara, CA, USA) for quality control. Sequencing was conducted on the NovaSeq 6000 (Illumina, CA, USA) with single-end 75 bp reads to obtain a minimum of 5M reads per sample. Quality of the FASTQ sequencing reads was assessed using FastQC. Reads were mapped to the reference genomes *D. rerio* (Build danRer11) using the BlueBee Genomics Platform (BlueBee, Rijswijk, The Netherlands).

After obtaining the raw reads, the sequencing data were then analyzed according to previous studies [29,31]. Differential gene expression between the control and exposed samples was analyzed using DEseq2 (GenePattern; Broad Institute, Cambridge, MA, USA). To minimize the false negative rate, genes with a raw *p*-value < 0.05 and an absolute log2 fold change ≥ 0.75 were defined as differentially expressed genes (DEGs). The (DEGs) across all exposure groups were uploaded into Ingenuity Pathway Analysis software (IPA; QIAGEN Bioinformatics, Redwood City, CA, USA) using Ensembl gene ID and converted to human orthologs. The implicated biological processes and canonical pathways associated with the DEGs were determined by the right-tailed Fisher’s Exact Test with a *p*-value < 0.05.

## 3. Results

We conducted dose–response studies to identify transcriptomic and phenotypic responses in 2-year-old zebrafish following a 5-day acute Pb exposure. Behavioral changes in the aged zebrafish after exposures are shown in Figure S1. We found fish exposed to 10 and 100 µg/L Pb exhibited the least distance moved compared to the control and other exposed groups, while fish exposed to 10,000 µg/L of Pb moved slightly longer distances (Figure S1A). Among the anxiety-like behaviors (Figure S1B–E), we observed that the average time spent at the top and bottom of the tank (Figure S1B) and the distances moved at these locations (Figure S1C) had a corresponding activity pattern. The activity at the top of the tank was highest for the 10 and 100 µg/L exposure groups, followed by the 1 and 1000 µg/L groups, and then the control group. On the other hand, zebrafish exposed to 10,000 µg/L moved the longest distance and spent an average of 94% of their time at the bottom of the tank, indicating the most apparent anxiety-like behavior among all groups. Although 100 µg/L-exposed fish moved the shortest distances across the whole test period, they spent an average of 60% of their time at the bottom, indicating a more even movement between the top and bottom of the tank. There were few cases of erratic movements and freezing bouts across Pb exposure levels. While a non-monotonic response in activity was observed across all exposure conditions, no statistically significant differences were found among them.

**Figure 1:**
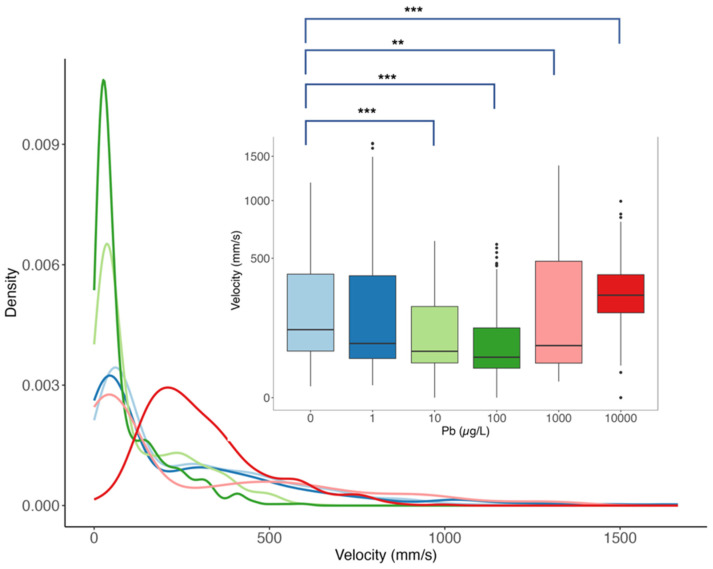
Velocity distribution of 2-year-old *Danio rerio* after 5 days of Pb exposure. Colors represent exposure concentrations: 0 (light blue), 1 (dark blue), 10 (light green), 100 (dark green), 1000 (pink), and 10,000 (red). The subplot displays a box plot indicating the median, quartiles, and outliers of velocity for each exposure. Pairwise Dunn–Bonferroni post hoc test pair test compares exposure groups with control (** *p*-value < 0.01, *** *p*-value < 0.001).

When comparing the velocity distribution across Pb concentrations (Figure 1), with velocity integrated as 3 s interval per fish replicate, significant differences were observed in the distribution of velocity in all exposure groups compared to the control group (*p* < 0.01), except for the 1 µg/L level. We noted a decreasing trend in velocity distribution from the control group to 100 µg/L exposure, with the control mode velocity of 6 mm/s dropping to 3 mm/s at 100 µg/L (*p* < 0.01). At 10,000 µg/L exposure level, the velocity distribution shifted drastically to the right, indicating a mode velocity of 20 mm/s. This hyperactive movement at 10,000 µg/L corresponded with the anxiety-like behavioral results.

**Figure 2:**
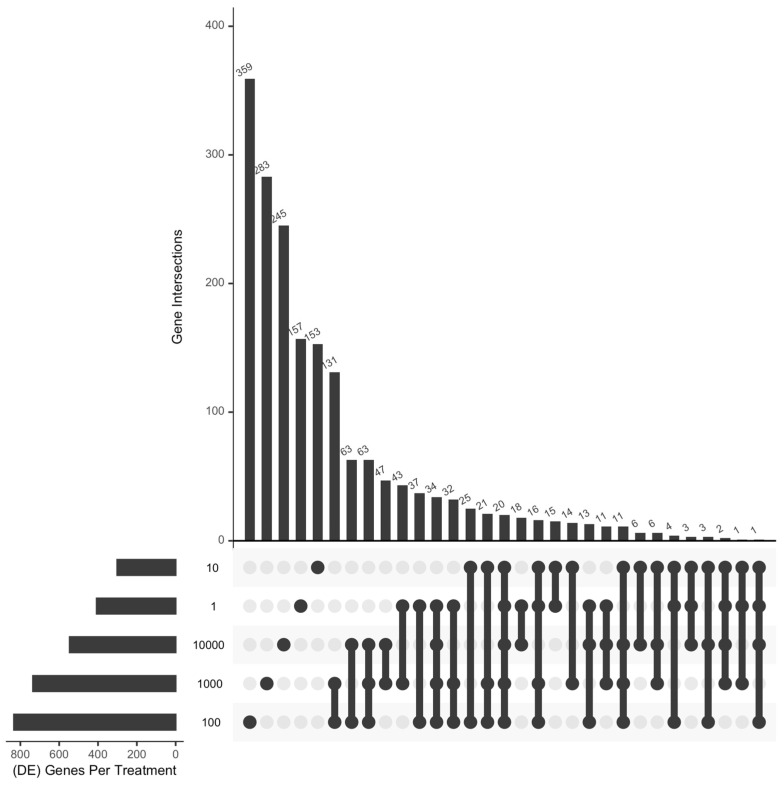
Upset plot of the frequency distrubition of differentially expressed genes (DEGs) and their intersection in 2-year-old *Danio rerio* after 5 days of Pb exposure. The upset plot was generated using the package UpSetR in R software (version 4.2.3).

Summed across all exposure levels, a total of 2822 differentialy expressed genes (DEGs) were reported in aged zebrafish after Pb exposure, with 65% identified as unique to a single condition (Figure 2, Table S1). Twenty genes were differentially expressed across all Pb exposure levels (Table 1). Among those, five were DEGs relevant to cytoskeletal regulation, including tubulin genes (*tuba1a*, *tubb2a*, *ttll1*), a tubulin family regulator (*stmn2*), and a neurofilmament gene (*nefm*). Another three genes were implicated in mitochondrial dysfunctions, including two mitochondrial-localizing genes (*ndufs3* and *pink1*) and one Na^+^/K^+^ transporting gene (*atp1a3*). The rest of the downregulated genes were implicated in various functions, including heat shock protein expression (*hsp90aa1* and *eef1a2*), glucose metabolism (*gapdhs*), and biosynthesis (*fabp2*). The remaining eight DEGs had a more variable pattern of response within the same class of gene ontology: for example, two genes that encoded long-term potentiation regulators were affected differently by Pb exposure, with one upregulated (*hcn1*) and one downregulated (*xbp1*). Three genes relevant to gene modification were similarly affected, including a post-translation factor (*oga*) and a chromatin configuration factor (*hmgn2*) that were upregulated, and a post-transcriptional factor (*rsrp1*) that was downregulated. Likewise, one DEG in the category of DNA damage and stress response was upregulated (*isg15*) and the other was downregulated (*rfwd3*).

**Figure 3:**
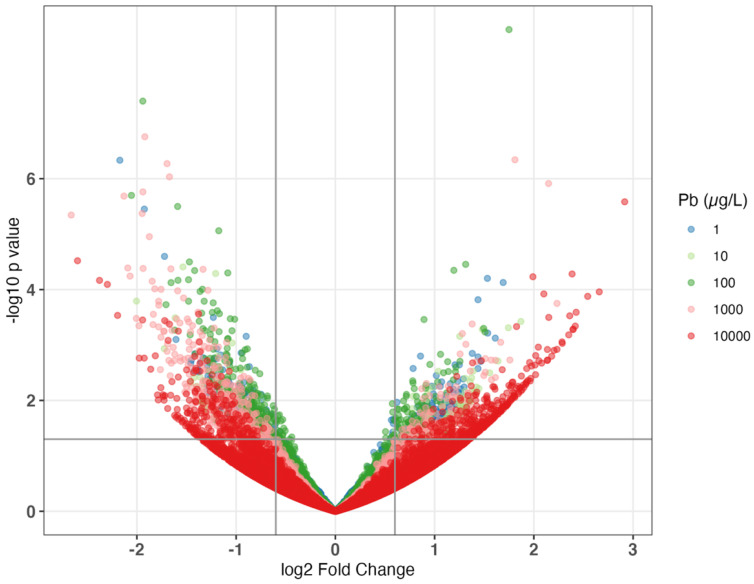
Volcano plots of differentially expressed genes (DEGs) in 2-year-old *Danio rerio* after 5 days of Pb exposure. Grey lines indicate a threshold of *p*-value < 0.05 and an absolute log2 fold change at 0.75. Colors represent exposure concentrations: 1 (dark blue), 10 (light green), 100 (dark green), 1000 (pink), and 10,000 (red). Individual volcano plots along with the number of up- and downregulated DEGs and their corresponding log2 fold changes for each treatment can be found in Figure S2.

Among all exposure levels, 100 µg/L Pb had the highest number of dysregulated genes at 833, followed by 1000 and 10,000 µg/L with 735 and 606 DEGs, respectively (Figure 2). Nevertheless, we observed an increasing dose–response relationship between Pb level and the average absolute log2 fold change of DEGs (Figure 3 and Figure S2). Exposure to 1000 or 10,000 µg/L induced the most substantial downregulation among all concentrations, both exhibiting an average log2 fold change of −1.3. Exposure to 10,000 µg/L showed the highest upregulation, with an average log2 fold change of 1.5. Thus, although our intermediate dose (100 µg/L) altered the most DEGs, the average magnitude of change was less than that observed with higher doses (1000 and 10,000 µg/L).

**Figure 4:**
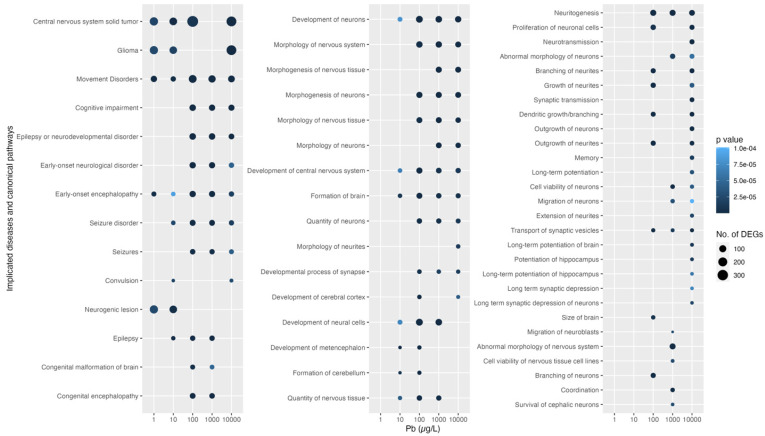
Ingenuity Pathways Analysis for transcriptomic changes implicated in neurological diseases and canonical pathways after 5-day Pb exposure in 2-year-old *Danio rerio*. Dot symbols indicate the number of differentially expressed genes (DEGs) associated with each implication, with color representing the *p*-value.

Using IPA, we identifed the diseases and dysfunctions associated with these transcriptomic changes, primarily occurring at a Pb exposure equal to or greater than 100 µg/L (Figure 4). The number of affected neurologic pathways increased with concentration, with 10,000 ug/L showing the largest number (45 of of the 58 identified). Movement disorders were implicated at every concentration tested. Cognitive impairment, neurodevelopmental disorder, and seizures were implicated at concentrations ranging from 100 to 10,000 µg/L. Specifically, at 100 µg/L, most transcriptomic changes were associated with the development, morphogenesis, growth, and proliferation of neurons and neural cells. The transcriptomic changes at 10,000 µg/L were exclusively associated with alterations of synaptic transmission, extension of neurites, and long-term potentiation.

**Figure 5:**
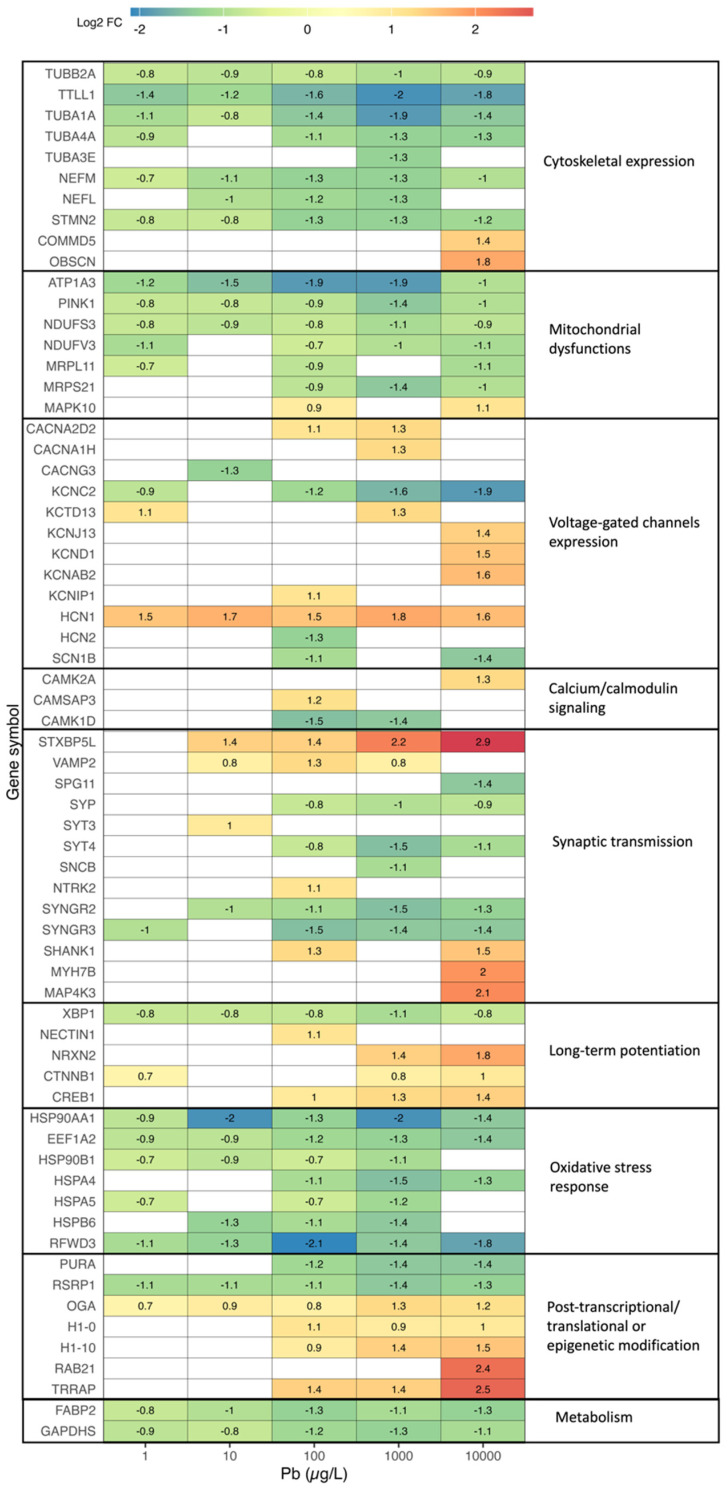
Highly differentially expressed genes (DEGs) relevant to aging-related functions in 2-year-old *Danio rerio* after 5-day Pb exposure, including non-monotonic transcriptomic responses. Colors represent the log2 fold changes (log2 FC) of each DEG.

In Figure 5, we present the log2 fold changes of highly expressed DEGs and categorize them into nine biological functions. Five of these functions fall under neurotransmission, including voltage-gated channel expression, calcium/calmodulin signaling, cytoskeletal regulation, synapse transmission, and long-term potentiation. Additionally, we identified four functions related to oxidative stress, including oxidative stress response, mitochondrial dysfunction, heat shock protein expression, and DNA damage. Various pathways related to gene regulation, such as post-transcription, post-translation, and epigenetics, as well as functions linked to metabolism, were also observed.

## 4. Discussion

An important observation from our study is the contrasting locomotor activity trend exhibited by aged male zebrafish at low (≤100 µg/L) and high (1000–10,000 µg/L) Pb levels. We found that aged zebrafish showed hypoactive behaviors following exposure to Pb levels less than or equal to 100 µg/L but became hyperactive after exposure to 1000–10,000 µg/L. Previous studies have predominantly demonstrated that long-term exposure to Pb concentrations under 100 µg/L reduces locomotor activity in adult fish [18,32]. Moreover, early-life exposure to parts per billion (ppb) Pb exposure levels can induce behavioral changes in later adult life, including memory deficits, slow escape responses, reduced movement, and impaired exploratory abilities [16]. While only a few studies have shown inconsistent patterns of hyperactive behavior following ppb-Pb exposure levels [16,33], developmental mouse models have shown that Pb dosages up to parts per million (ppm) levels have been associated with hyperactive responses and anxiety-like behaviors. These behaviors can manifest after long-term exposure during childhood [34,35,36,37]. Our study is the first to demonstrate the U-shape of Pb dose and movement response in aged zebrafish following acute exposure, shedding new light on the potential behavioral effects of Pb in the aging population.

As 10,000 µg/L Pb levels exclusively induced hyperactive responses, our particular interest lay in transcriptomic changes occurring solely at this concentration, including synaptic transmission and long-term potentiation. Synaptic transmission requires the regulation of calcium ions across cell membranes through voltage-gated channels, the activation of calmodulin and calmodulin kinases, the phosphorylation of synaptic vesicle-associated proteins, and the release of neurotransmitter vesicles and neurotransmitters to relay signals from the presynaptic to the postsynaptic membrane [38]. In our study, exposure to 10,000 µg/L induced overexpression of several genes involved in the abovementioned process, including a calmodulin kinase gene, *camk2a*, which encodes calcium/calmodulin dependent protein kinase II alpha (CaMK2a), and *stxbp5* encoding syntaxin-1-binding protein 5 (Stxbp5). CaMK2a is a crucial enzyme that phosphorylates and activates proteins for neurotransmitter synthesis [39] and long-term potentiation at the hippocampus [40]. The elevation of CaMK2 activity in the brain region was associated with juvenile male rats exhibiting behaviors of attention-deficit/hyperactivity disorder (ADHD) [41]. Induction of STXBP5 has been found to impair spatial learning and memory by inhibiting transmitter release and reducing synaptic transmission in hippocampal neurons of adult mice [42]. Another regulator, *commd5*, was solely upregulated at 10,000 µg/L. The induction of COMMD5 can alter its interaction with acttin filaments and microtubules, eventually destabilizing microtubule dynamics and neuronal activity [43].

Several voltage-gated channel genes were also solely altered at 10,000 µg/L, including the upregulation of potassium voltage-gated channel genes *kcnd1* and *kcnab2*, as well as downregulation of sodium voltage-gated channel beta subunit 1, *scn1b*. Accessory subunits of sodium channels can control neuronal excitability by interacting with the expression of potassium channel complexes [44]. Genes involved in the transport of synaptic components to the synapse were also altered, such as the downregulation of *myo5a* encoding a filamentous-actin motor protein [45] and the upregulation of *snap47* encoding synaptosome associated protein 47 [46]. These alterations can disrupt neuronal ionic flow, contributing to hyperexcitability and potentially triggering motor dysfunction or seizure activity [47].

At exposure levels of 100 µg/L, where locomotion activity was lowest and the number of DEGs was highest, our analysis revealed that non-monotonic dose response transcriptomic changes were predominantly associated with disturbance in nervous system development. Contrary to the previous belief that neurodevelopmental activity only occurred early in life, it is now understood that neurogenesis and cell differentiation continue to take place in the mature brains of all vertebrates [48], particularly in zebrafish up to 36 months of age [49]. Thus, alteration in the expression of neurodevelopmental genes would be expected to occur throughout the aging process [50].

Synpatogenesis naturally deteriorates with the aging process, leading to a significant decline in the expression of key pre- and post- synaptic proteins expression in aged zebrafish [51]. A similar phenomenon is observed in normal human aging and Alzheimer’s disease, where multiple brain regions undergo extensive downregulation in synaptic gene expression [52]. In our study, we found that synapse-associated DEGs were predominantly downregulated by Pb exposure: *syt4* (encoding synaptotagmin 4) and *syp* (encoding synaptophysin) at 100–10,000 µg/L, as well as *syngr2* (encoding synaptogyrin 2) and *syngr3* (encoding synaptogyrin 3) at 10–10,000 µg/L. Our findings underscore that Pb exposure may further exacerbate the downregulation of synaptic genes during aging.

Genes encoding microtubules and neurofilaments, which constitute the neuronal cytoskeleton, were also found to be downregulated due to Pb exposure. These genes include four microtubulin genes (*tuba1a*, *tubb2a*, *tuba4a*, and *tuba3e*) and two neurofilament encoding genes (*nefm* and *nefl)*. The cytoskeleton is essential for maintaing neuronal structure and is involved in various cellular processes, such as migration, proliferation, and degeneration [53]. Neurofilaments can release fragments from neurons into the bloodstream after neuronal damage or neurodegeneration [54]. Among these dysregulated genes, *tuba1a* encodes the most common α-Tubulin isotype and is highly expressed during early neuronal development to assemble microtubules for neurite extension but is significantly downregulated in the adult brain [55]. On the other hand, *tubb2A* and *tuba4A* are β-tubulin genes with consistently high expression levels in the brain into adulthood [56] and show substantial increases with age [57]. Mutations in *tuba1a*, *tubb2a*, and *tuba4a* have all been linked to a group of clinical neurodevelopmental disorders known as tubulinopathies [58,59,60]. Deficits in these tubulin-associated genes can impair synaptic maintenance [61] and destabilize microtubule dynamics [59,60], thus leading to motor dysfunctions. The neuronal cytoskeleton system serves diverse functions throughout the aging process, and its transcriptome can be significantly impacted by Pb exposure in the aged population, potentially contributing to our non-monotonic outcome of decreased velocity in 100 µg/L Pb-exposed fish.

Our study found some key genes involved in the regulation of neurotransmission gene response at different exposure levels. One DEG, *oga*, was upregulated across all Pb exposure concentrations. *OGA* encodes the post-translational modifier of calmodulin protein kinases and other neuronal or synaptic proteins [62], including the cyclic AMP-response element binding protein (CREB) encoded by *CREB. Creb* was also upregulated at increasing Pb exposure levels ranging from 100 to 10,000 µg/L in aged zebrafish. Persistent activation of *CREB* has been identified in the cerebral cortex of patients with seizure disorder [63]. Given that CREB is downstream of CAMK2a and is activated by calcium influx and cAMP signal, changes in *creb* expression may potentially affect the transcription of downstream genes responsible for synaptic plasticity, learning, and memory [63,64]. Another DEG, *pura*, was downregulated at 100 to 10,000 µg/L. *PURA* encodes a purine-rich DNA and RNA binding protein that is involved in the transport of nucleic acids within neuronal tissue, associated with myosin *MYO5a* and kinesin *KIF5* [65]. Suppression of *pura* may disrupt neurogenesis and alter the cytoskeleton of neurons as well as synapse formation [66]. Another key gene of neuron-specific synaptic signaling, *stmn2*, was repressed at all Pb concentrations. The repression of *stmn2* has been found to impair synaptic trafficking and induce neuron loss in mice [67]. In fact, according to network analysis, human *stmn2* has been identified as one of the key genes associated with the pathogenic pathways of Parkinson’s disease [67]. It is possible that Pb-induced transcriptomic changes disrupt the regulation of neurotransmission pathways, ultimately reducing memory and learning functions that rely on long-term potentiation, as well as leading to other neurodegenerative diseases.

In addition to the disruption and deterioration of the abovementioned neurological pathways, aging leads to the accumulation of other various biological dysfunctions over time, such as the accumulation of oxidative stress, mitochondrial dysfunction, and DNA damage [68]. Since cellular processes in neurons, including synaptic plasticity and neurotransmitter synthesis, demand high-energy sources, the brain is one of the organs that heavily relies on mitochondrial energy production through oxidative phosphorylation [69]. However, the aging process leads to a decrease in respiratory capacity [70], ATP synthesis [71,72], and mitochondrial membrane potential [71], and thus the disruption of normal mitochondrial function becomes more substantial in old age. In our study, we found that Pb-induced transcriptomic changes were also implicated in mitochondrial dysfunction, with a higher number of DEGs at 100 µg/L compared to other exposure levels. Most of these genes were downregulated, encodeding ATPase Na^+^/K^+^ transporting subunits (*atp1a1*, *atp1a3*, *atp5f1b*), NADH–ubiquinone oxidoreductase subunits (*ndufs3*, *ndufv3*), mitochondrial ribosomal proteins (*mrpl11*, *mrps21*), and NADH dehydrogenase subunits (*mt-nd2*, *mt-nd3*). In particular, mutations of *atp1a3* in human patients were implicated in multiple neurological disorders involving abnormal movements, altered awareness, and autonomic dysfunction [73]. Human studies have also found reduced expression of mitochondrial genes in the blood of individuals with Alzheimer’s disease and mild cognitive impairment [74]. However, as mitochondrial gene expression is tightly regulated by approximately 100 oxidation phosphorylation protein subunits encoded by both nuclear and mitochondrial genomes [75], further studies are needed to determine if Pb-induced transcriptomic changes in aged populations can lead to detrimental effects in mitochondria.

Nevertheless, the accumulation of oxidative stress during aging can result from dysfunctional mitochondria, which produce elevated levels of reactive oxygen species (ROS) [76], and ROS naturally produced from other organelles during aging [77]. Heat shock protein expression is a contributing mechanism of detoxification to mitigate oxdiative damage [78]. However, age-dependent studies have shown that the expression of several heat shock proteins decreases in old age in various organisms, including *Caenorhabditis elegans* [79], zebrafish [80], rats [81], and human subjects [82]. In our study, the majority of heat shock protein DEGs were downregulated following Pb exposures in aged zebrafish, including HSP90 (*hsp90aa1*, *hsp90b1*), HSP70 (*hspa4* and *hspa5*), and HSPB gene families (*hspb6*). The suppression of *hsp70* in adult zebrafish was also found in a previous study with Pb exposure levels ranging from 1 to 100 µg/L [32]. Specifically, HSP70 plays a crucial role in responding to stress, such as neutralizing apoptosis pathways [83] and processing the misfolded proteins in brains affected by Alzheimer’s or Parkinson’s diseases [84,85]. Suppression of heat shock proteins induced by Pb exposure may impair the protective mechanisms in the brain, potentially increasing the susceptibility of the aged brain to neurodegenerative disorders.

Finally, we observed transcriptomic changes in neuro-related epigenetic pathways. Previous studies have highlighted the role of epigenetic mechanisms in various neurological functions and diseases [86], and these mechanisms can be influenced by metal exposure [87]. Developmental exposure to Pb levels ≤ 500 µg/L has been found to reduce methyltransferase activity, consequently leading to a decrease in global methylation levels in zebrafish [88]. Given the typical age-associated decline in total genomic DNA methylation [89], the effects of developmental Pb exposure at ppm levels were compared to those of the normal aging process in the aged rodent brain [90]. It was found that developmental Pb exposure can induce a more substantial repression of total gene expression compared to the normal aging process [90]. Moreover, our previous study found transgenerational impacts due to developmental Pb exposure in the unexposed F2 generation of zebrafish, possibly through chromatin remodeling and histone modification [29]. In this study, we identified several neurological genes that may be regulated by DNA methylation, such as *shank1* encoding a synaptic scaffolding protein in both epileptic rats and epilepsy patients [91], as well as mitochondrial genes *mt-nd2* and *mt-nd3* in human brains [92]. Regarding histone modification, we detected the upregulation of *trrap* by Pb exposure at 100 to 10,000 µg/L, which may disrupt neurogenesis by impairing the recruitment of histone acetyltransferases and altering the fate decision of neural progenitor cells [93]. Another two DEGs encoding linker histones (*h1-0* and *h1-10*) were also suppressed at 100 to 10,000 µg/L. Intrestingly, *h1-0* was also found to be dysregulated in the F2 generation of zebrafish following ancestral Pb exposure [29].

In summary, our study sheds light on the complex interplay between Pb exposure and neurological pathways in the aged population. We are the first to demonstrate a U-shape response in both movement patterns and transcriptomic changes in the aged population acutely exposed to Pb. Pb levels ≥ 100 µg/L induced the highest number of DEGs in the aged brain, with the most substantial downregulation at Pb ≥ 1000 µg/L and the most pronounced upregulation at 10,000 µg/L. These DEGs were associated with various neurological diseases, including cognitive impairment, early-onset neurological disorder, neurodevelopmental disorder, and movement and seizure disorders. Notably, several of these DEGs, including *atp1a3*, *mt-nd2*, *mt-nd3*, *hsp70*, *stmn2*, *tuba1a*, *tubb2a*, and *tuba4a*, have been found to be dysregulated in human clinical cases, highlighting the relevance of our study to Pb-induced effects in humans. While the zebrafish model may not fully capture the complexity of mammalian brain structures and behaviors, the DEGs identified provide valuable targets for future research in mammalian models.

Furthermore, our findings unveiled the potential impact of Pb on the aging process. Aging is known to lead to significant declines in gene and protein expression related to synaptic transmission, cytoskeletal regulation, oxidative stress response, and epigenetic regulation. While the alterations in these biological functions are closely linked to the development of neurodegenerative diseases, we demonstrated that Pb exposure further downregulates genes associated with these biological functions and affects their post-transcriptional or translational modification processes, as well as epigenetic mechanisms. These findings provide additional evidence underscoring that neurodegenerative diseases are not solely infuenced by individual factors but are also impacted by environmental exposures. Since our study focused on the potential mechanisms behind male-specific susceptibility to Pb, future studies should conduct comparative analysis of behavioral and transcriptomic effects in both sexes. Given our previous findings that the behavioral and transcriptomic outcomes of early-life Pb exposure can also persist transgenerationally to unexposed adult F2 descendants, the persistence of Pb-induced epimutations and their link with adverse neurobehavioral outcomes during neurosensitive windows, both early development and geriatric, requires further investigation.

## Figures and Tables

**Table 1:** Twenty overlapped differentially expressed genes (DEGs) across all Pb levels.

Gene Symbol	Gene Name
*TUBA1A*	tubulin alpha 1a
*TUBB2A*	tubulin beta 2A class IIa
*TTLL1*	TTL family tubulin polyglutamylase complex subunit L1
*STMN2*	stathmin 2
*NEFM*	neurofilament medium chain
*NDUFS3*	NADH–ubiquinone oxidoreductase core subunit S3
*PINK1*	PTEN induced kinase 1
*ATP1A3*	ATPase Na^+^/K^+^ transporting subunit alpha 3
*HSP90AA1*	heat shock protein 90 alpha family class A member 1
*EEF1A2*	eukaryotic translation elongation factor 1 alpha 2
*GAPDHS*	glyceraldehyde-3-phosphate dehydrogenase, spermatogenic
*FABP2*	fatty acid binding protein 2
*HCN1*	hyperpolarization activated cyclic nucleotide gated potassium channel 1
*XBP1*	X-box binding protein 1
*OGA*	O-GlcNAcase
*HMGN2*	high mobility group nucleosomal binding domain 2
*RSRP1*	arginine and serine rich protein 1
*ISG15*	interferon-stimulated gene 15 ubiquitin-like modifier
*RFWD3*	ring finger and WD repeat domain 3
*CALY*	calcyon neuron-specific vesicular protein

## Data Availability

All data are included in this document and Supplementary Materials.

## Supplementary Materials

The following supporting information can be downloaded at: https://www.mdpi.com/article/10.3390/toxics12100745/s1, Figure S1: Behavioral changes of Pb exposure in zebrafish in the novel tank diving test; Figure S2: Individual volcano plots of differentially expressed genes (DEGs) for each Pb exposure; Table S1: Differentially expressed genes for all concentrations compared to the control.
