# Supplementary material for: Implications of Lead (Pb)-Induced Transcriptomic and Phenotypic Alterations in the Aged Zebrafish (Danio rerio)
Source: Toxics. 2024 Oct 14;12(10):745. doi: 10.3390/toxics12100745 (PMC11511149; doi:10.3390/toxics12100745)
Supplement: Supplementary file 1 [file toxics-12-00745-s001.zip › DEGs Appendices figures.pdf]

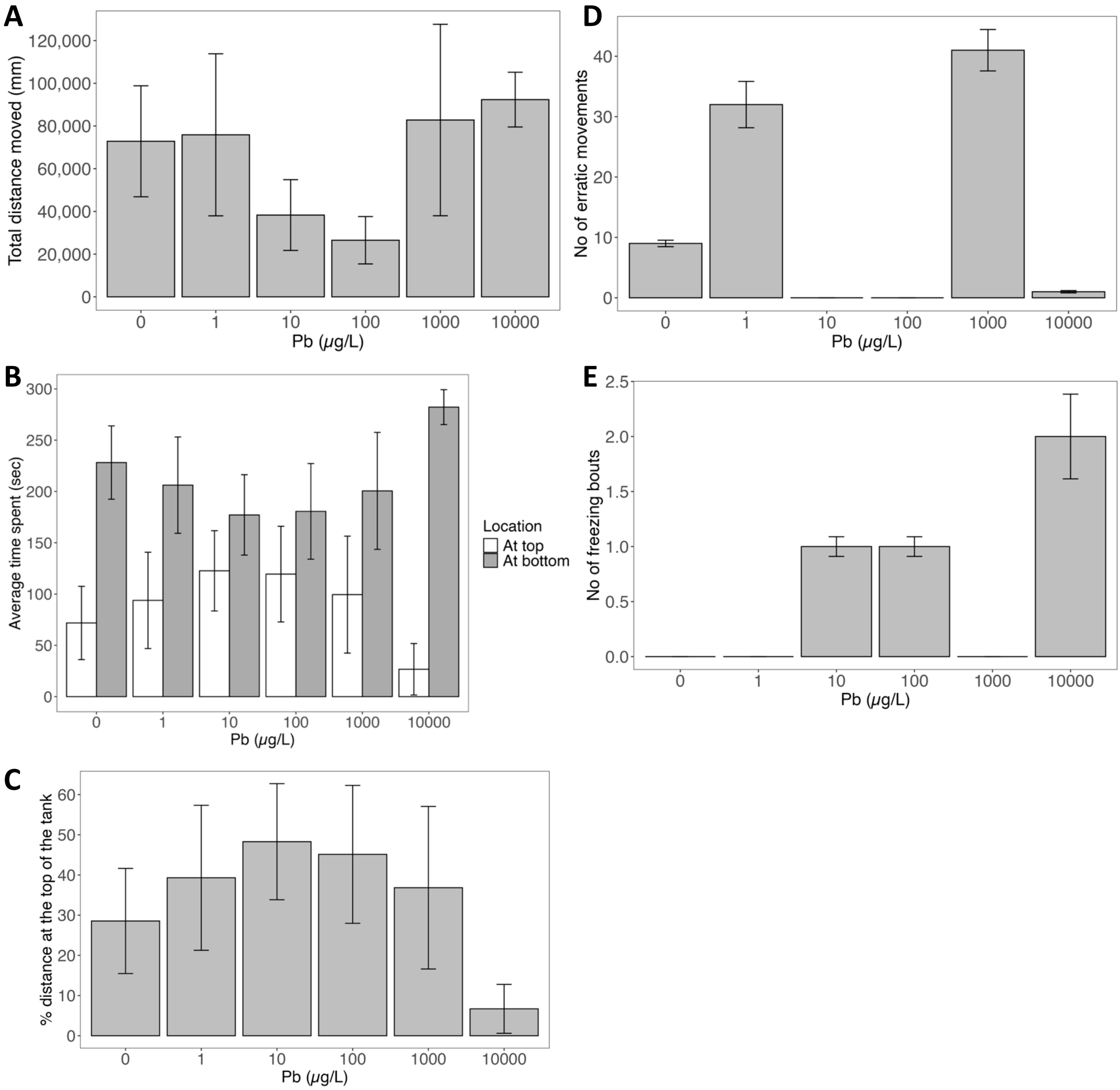

Figure S1. Behavioral changes of Pb exposure on zebrafish in the novel tank diving test, including (a) Total distance moved; (b) Time spent at the top and bottom of the tank; (c) % distance at the top of the tank; (d) Number of erratic movements; and (e) Number of freezing bouts. Data are presented as mean ± SEM.

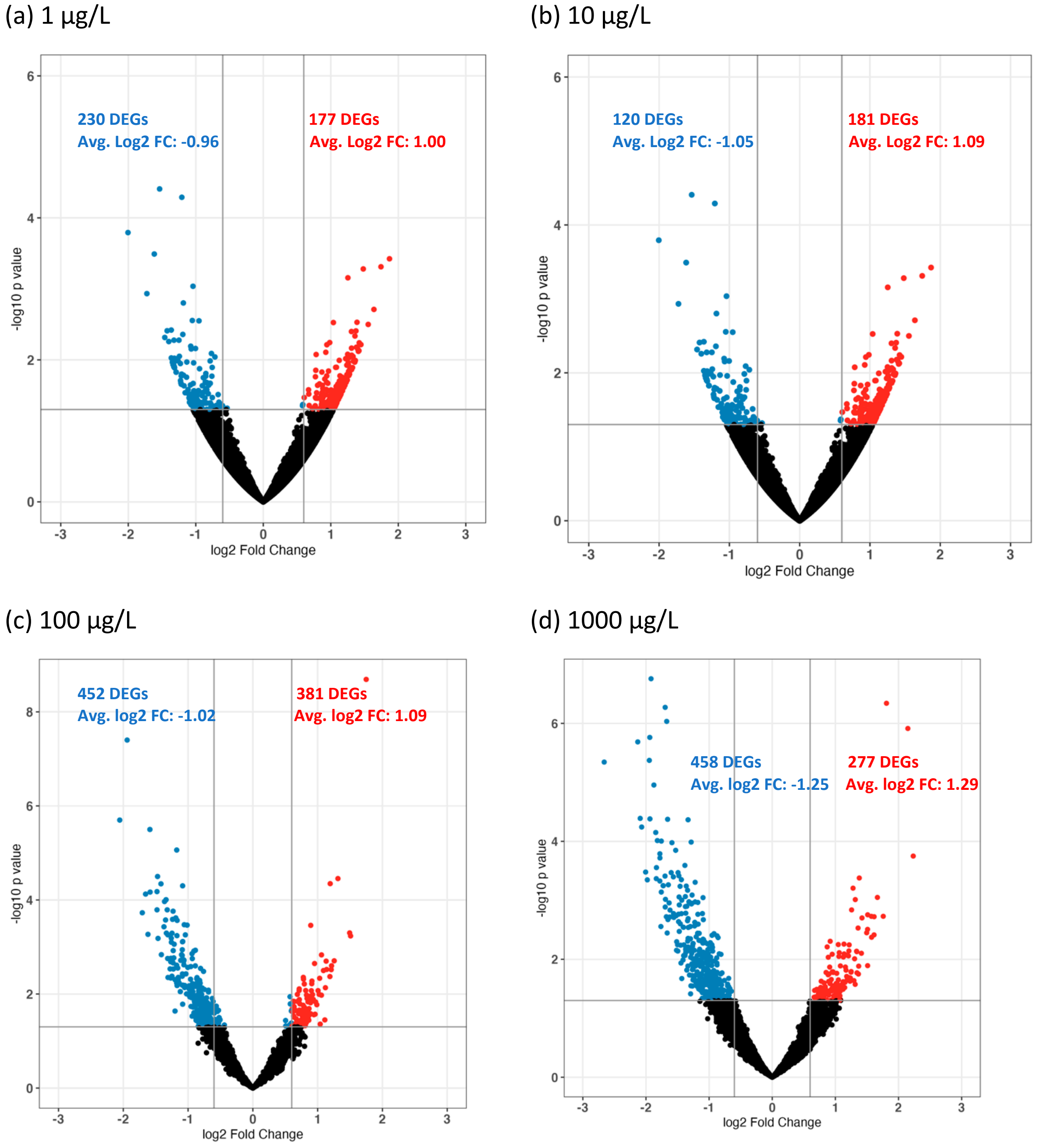

(a) 1 µg/L
230 DEGs
Avg. Log2 FC: -0.96
177 DEGs
Avg. Log2 FC: 1.00
-log10 p value
log2 Fold Change
(b) 10 µg/L
120 DEGs
Avg. Log2 FC: -1.05
181 DEGs
Avg. Log2 FC: 1.09
-log10 p value
log2 Fold Change
(c) 100 µg/L
452 DEGs
Avg. log2 FC: -1.02
381 DEGs
Avg. log2 FC: 1.09
-log10 p value
log2 Fold Change
(d) 1000 µg/L
458 DEGs
Avg. log2 FC: -1.25
277 DEGs
Avg. log2 FC: 1.29
-log10 p value
log2 Fold Change

(e) 10000 μg/L

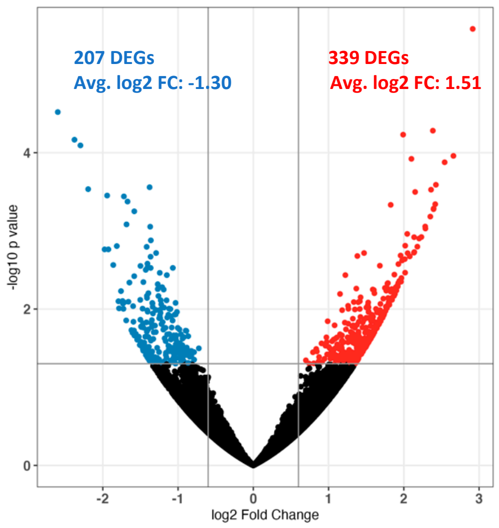

Figure S2. Individual volcano plots of differentially expressed genes (DEGs) for each Pb exposure. Grey lines indicate a threshold of *p* value < 0.05 and an absolute log2 fold change at 0.75. Colors represent upregulated (red) and downregulated (blue) DEGs, with texts indicating the number of DEGs and their corresponding log2 fold changes.
